# Supplementary material for: Homologous and Heterologous Vaccination Regimens with mRNA and rVSV Platforms Induce Potent Immune Responses Against SFTSV Glycoprotein
Source: Viruses. 2025 Aug 8;17(8):1095. doi: 10.3390/v17081095 (PMC12390526; doi:10.3390/v17081095)
Supplement: Supplementary file 1 [file viruses-17-01095-s001.zip › Supplemental Figure 1 Copy Editing.pdf]

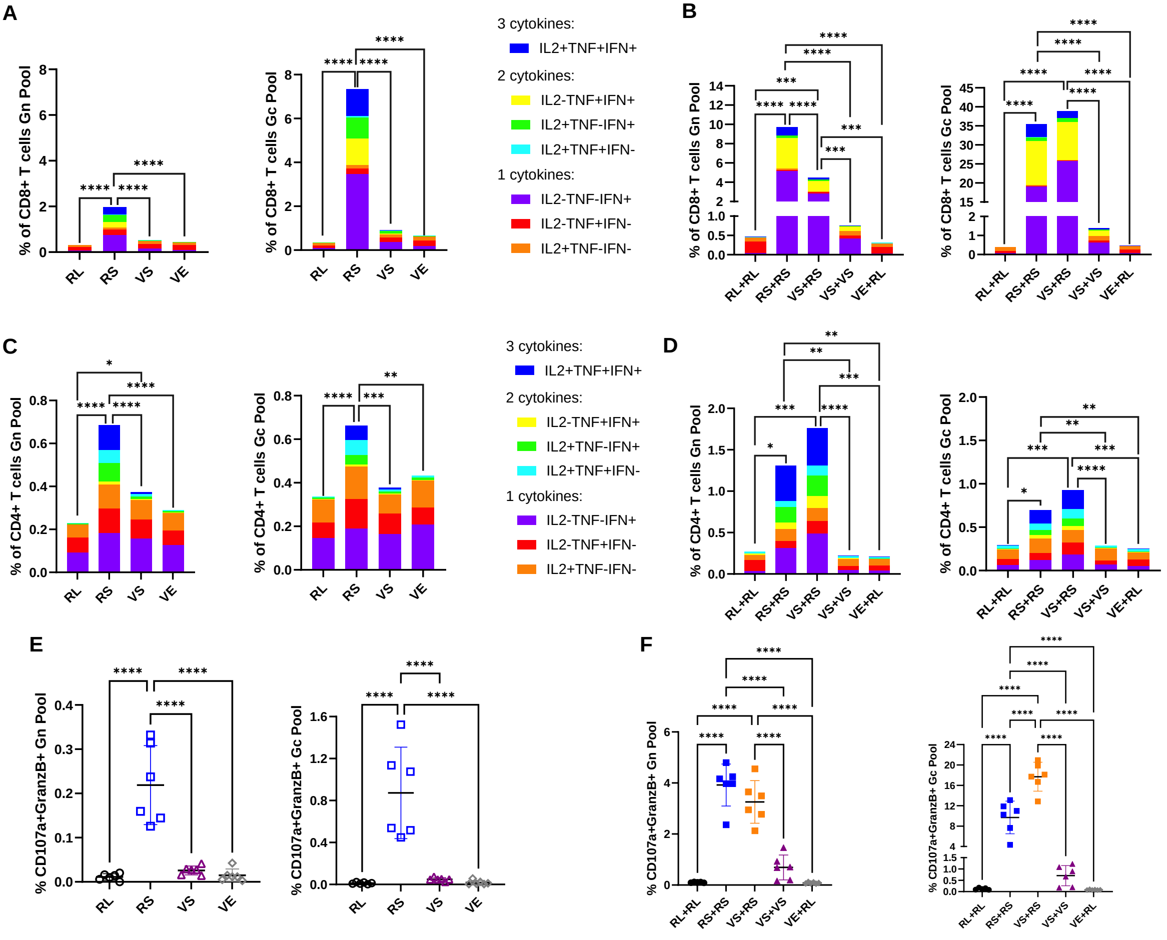

A
% of CD8+ T cells Gn Pool
% of CD8+ T cells Gc Pool
RL
RS
VS
VE
3 cytokines:
IL2+TNF+IFN+
2 cytokines:
IL2-TNF+IFN+
IL2+TNF-IFN+
IL2+TNF+IFN-
1 cytokines:
IL2-TNF-IFN+
IL2-TNF+IFN-
IL2+TNF-IFN-
B
% of CD8+ T cells Gn Pool
% of CD8+ T cells Gc Pool
RL+RL
RS+RS
VS+RS
VS+VS
VE+RL
C
% of CD4+ T cells Gn Pool
% of CD4+ T cells Gc Pool
D
% of CD4+ T cells Gn Pool
% of CD4+ T cells Gc Pool
E
% CD107a+GranzB+ Gn Pool
% CD107a+GranzB+ Gc Pool
F
% CD107a+GranzB+ Gn Pool
% CD107a+GranzB+ Gc Pool
