# Supplementary figures and images for: Homologous and Heterologous Vaccination Regimens with mRNA and rVSV Platforms Induce Potent Immune Responses Against SFTSV Glycoprotein
Source: Viruses. 2025 Aug 8;17(8):1095. doi: 10.3390/v17081095 (PMC12390526; doi:10.3390/v17081095)

**A**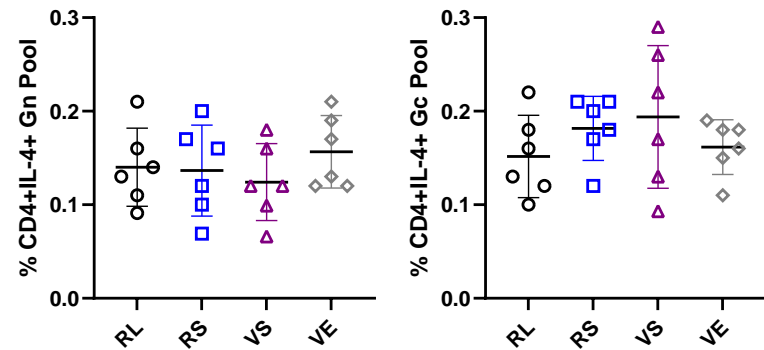**B**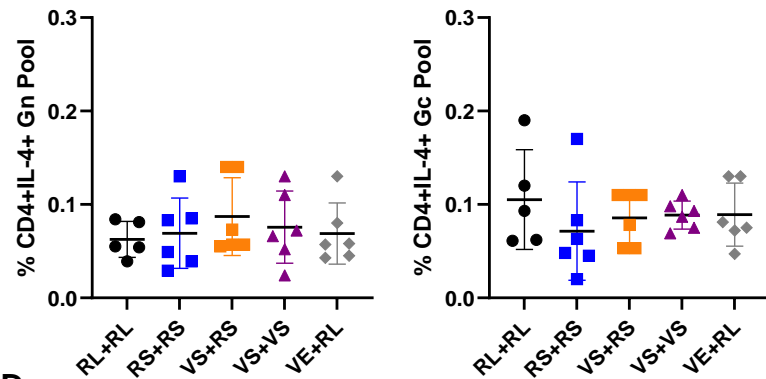**C**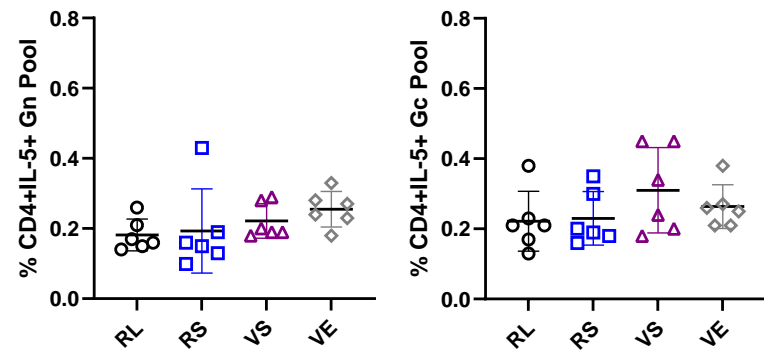**D**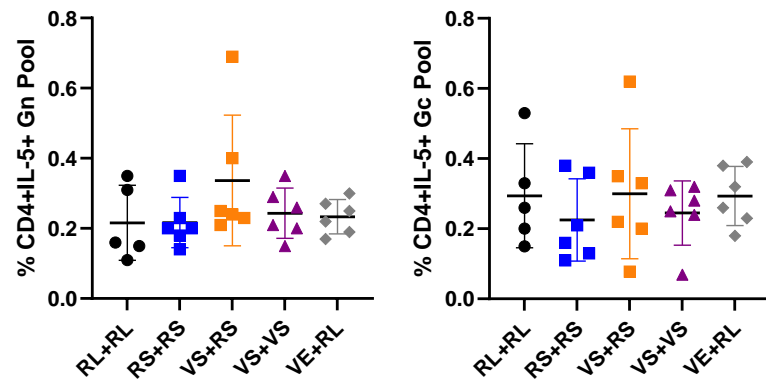

Supplement: Supplementary file 1 [file viruses-17-01095-s001.zip › Supplemental Figure 2.pdf]
